# A six-pronged approach to manage a *Plasmodium vivax* outbreak in a low- and middle-income country on the road to malaria elimination

**DOI:** 10.1371/journal.pntd.0013970

**Published:** 2026-02-13

**Authors:** Mergiory Labadie-Bracho, Gaitree K. Baldewsingh, Hélène Hiwat van Laar, Malti R. Adhin

**Affiliations:** 1 “Prof. Dr. Paul C. Flu” Institute for Biomedical Sciences, Paramaribo, Suriname; 2 Medical Mission Primary Health Care Suriname, Paramaribo, Suriname; 3 Ministry of Health Malaria Program, Ministry of Health, Paramaribo, Suriname; 4 Faculty of Medical Sciences, Department of Biochemistry, Anton de Kom Universiteit van Suriname, Paramaribo, Suriname; Ohio State University, UNITED STATES OF AMERICA

## Abstract

**Background:**

Malaria remains a global health concern, with *Plasmodium vivax* as predominant species in the Americas. The region has made notable strides in reducing malaria incidence, but challenges persist. Guiana Shield countries share similar malaria ecologies and vector dynamics, yet their progress toward elimination is quite different. Suriname, a low-middle income country, has pursued malaria elimination and became the first Amazonian country to receive malaria-free certification in June 2025. However, the reintroduction through imported cases from neighboring countries with higher endemicity remains a serious threat, as exemplified by the 2019 outbreak in Pelele Tepoe. The response strategy and operational measures implemented to address this *P. vivax* outbreak are described, along with key lessons for effective outbreak management.

**Methods:**

This descriptive study examines Suriname’s most recent *P. vivax* outbreak, using surveillance and intervention data from November 2019 to August 2021. It outlines the rationale for employing an adaptive, multi-pronged outbreak management strategy, details implementation of approaches, tailored to evolving outbreak circumstances and evaluates the effectiveness of subsequent interventions. Factors contributing to the outbreak’s prolonged duration, including COVID-19 pandemic impact are discussed, along with the resulting adaptations in the targeted responses.

**Results:**

The *P. vivax* outbreak lasted 22 months, resulting in 179 infections among 153 patients. Traditional strategies with corresponding interventions were introduced at onset, including “1-3-7” surveillance, community education and engagement and vector control. Continuous trend monitoring guided the adoption of additional less conventional and innovative measures, including mass screen-and-treat, mass drug administration (MDA), parasite genotyping and targeted treatment. Mass screen-and-treat was less effective in reducing parasite prevalence than MDA. Directly observed treatment reached 95.1% efficacy by day 180, however, relapses appeared to fuel the outbreak. Implementation of the “Radical Cure” marked the end of the outbreak.

**Conclusions:**

The described adaptive, integrated, six-pronged approach, combining conventional strategies with targeted accelerator methods and inclusive stakeholder engagement can effectively control outbreaks in low-endemic or near-elimination regions.

## Author summary

In 2004, Suriname still had the highest concentration of *Plasmodium falciparum* malaria cases in the Americas. Thanks to intensive control and surveillance efforts, the country reported zero indigenous cases since 2021. However, imported cases from neighboring high-endemic countries continue to pose a risk for reintroduction of malaria, as seen in the recent *P. vivax* outbreak in the Amerindian village of Pelele Tepoe. Our findings of the outbreak response demonstrate the success of a flexible, integrated six-pronged strategy, tailored to local needs and evolving conditions. A detailed description is provided of the implementation and effectiveness of each approach including the “1-3-7” surveillance, vector control and culturally sensitive community engagement. We also explain the rationale for implementing additional, less conventional targeted strategies with key measures as mass drug administration, molecular outbreak reconstruction, and new re-treatment protocols aimed at curtailing *P. vivax* relapses. Factors that contributed to the outbreak’s lengthy duration are discussed, including the COVID-19 pandemic impact and we share lessons learned, particularly the role of relapses and the importance of “Radical Cure” treatment. These insights offer valuable guidance for low-endemic countries or regions nearing malaria elimination, those facing reintroduction risks and regions struggling with persistent *P. vivax* relapses.

## Introduction

Malaria is a mosquito-borne infectious disease caused by the parasite *Plasmodium* and is still a major public health problem in many parts of the world. According to the World Health Organization (WHO) the worldwide number of malaria was estimated at 263 million cases in 2023. The region of the Americas (WHO classification), has been successful in reducing their malaria burden, as signified by a 65.4% reduction of malaria cases between 2000 and 2023. By 2023, the region reported 505,600 malaria cases, mostly due *to Plasmodium vivax* (72.1%) and only 342 deaths of the global number of 597,000 deaths [1].

Within the Americas, the Guiana Shield, which includes Guyana, Suriname, French Guiana, and parts of Venezuela (Bolivar and Amazonas states) and Brazil (Amapá Roraima and some Pará states), accounted for over half of *P. falciparum* and *P. vivax* cases in recent years [1].

With the current pace of global progress towards malaria elimination, countries will not reach the targets set by the Global technical strategy for malaria 2016–2030 and the Sustainable Development Goals (SDGs) for reduction of morbidity and mortality of malaria by 2030. To speed up this progress the WHO presented a detailed plan in 2021 [2] to guide nations in their quest to eliminate malaria.

Malaria endemic countries are encouraged to adapt their strategies, focus and interventions to the local context and challenges, with countries in the malaria control phase focusing on general system strengthening, and countries in the malaria elimination phase prioritizing monitoring and evaluation of each individual case [3].

It is worth noting that the countries in the Guiana Shield, albeit their similar malaria ecology and vector settings, are at different programmatic phases. Suriname, French Guiana and the state of Amapá in Brazil, on one hand recorded ample reductions in the number of reported cases over the past decade; while on the other hand, the state of Roraima in Brazil and Guyana exhibited a significant increase in malaria incidence, each reporting more than 20.000 cases in 2022 [4, 5] and even more than 30.000 cases in 2024 [6,7]. The highest malaria burden in the Americas was reported for Venezuela in 2022 [5] and for Colombia in 2024 [7].

Suriname was the country with the highest concentration of *Plasmodium falciparum* cases in the Americas in 2004 [8], but has recently received WHO certification for malaria elimination. Suriname achieved this milestone through rigorous control and monitoring measures, reducing indigenous malaria cases to zero. The last indigenous *P. falciparum* malaria case in the country was registered in 2018 [9] and the last indigenous *P. vivax* malaria case in October 2021 [10]. However, ongoing cross-border travel in the Guiana Shield regions, mainly by mobile migrant gold miners [11], results in cases of imported malaria in Suriname, which threaten to undo the national progress made toward malaria elimination. In 2019, cross-border case(s) from neighboring countries indeed led to the reintroduction of *P. vivax* malaria into Amerindian villages in the interior, sparking local outbreaks. The most recent malaria outbreak occurred in the Amerindian village Pelele Tepoe.

This report outlines the approach taken to address this outbreak and explains the rationale for implementing additional, less conventional strategies and methods to effectively contain this outbreak during the malaria elimination phase in Suriname.

Furthermore, it discusses the factors that contributed to the outbreak’s lengthy duration and shares key lessons learned, including strategies to prevent *P. vivax* relapses, which are fundamental for effective outbreak management in the future.

## Methods and results

### Ethics statement

This study is based on routine malaria surveillance information from the Ministry of Health in Suriname. The confidentiality of the study subjects was protected, and personal data was not shared. All patients provided informed verbal consent for malaria research at the time of enrollment in the National Malaria Gene Bank (NMGB). Ethical clearance for the NMGB was obtained by the National Ethics Committee within the Ministry of Health (VG007–08)”.

Surveillance was performed in compliance with relevant laws and guidelines from the Ministry of Health and the national ethics committee.

### Study design

This descriptive study of the latest *P. vivax* outbreak in Suriname presents malaria surveillance data and describes the use of conventional and innovative methods to manage the outbreak, along with the subsequent interventions and their effectiveness. These interventions were implemented during the outbreak from November 2019 to August 2021 by the Medical Mission Primary Health Care Suriname (MM), with support from the National Malaria Program (NMP), the Pan-American Health Organization (PAHO-Suriname) and under the supervision of the Malaria Elimination Taskforce (MET).

### Study settings and study population

Suriname is a small tropical, upper-middle income country on the northeastern coast of South America, part of the Guiana Shield and bordering Guyana, French Guiana, and Brazil. During endemic years, malaria transmission in Suriname was year‑round. The country’s coastal area, where the majority of the population lives, has been free of malaria since 1968. The primary malaria vector in the interior of Suriname is the anthropophilic *Anopheles darlingi*, which is a competent vector even in low mosquito densities [12]. In the last decade, malaria has also been almost non-existent in the Amazonian tropical rainforests in the interior of the country, where tribal communities of the Maroons (descendants of African slaves) and Amerindians live in settlements along the major rivers. However, a continuous influx of malaria is maintained through the uncontrolled cross border movement of people, mostly miners, from illegal gold mining settlements across the Amazon. In addition, Surinamese villagers also travel across borders for family visits. The Indigenous groups in southern Suriname belong to the Trio and Wayana tribes. The Trio tribe populates the northern Amazon Region on both sides of the Suriname-Brazil border, while the Wayana tribe is primarily found along the Tapanahony River in Suriname and on both banks of the Lawa River, at the Suriname-French Guiana border. Cross border visits are therefore a common phenomenon and inhabitants of areas within and across country borders sometimes congregate to partake in religious conferences.

Pelele Tepoe is an Amerindian village, located in the southern part of the country’s rainforest with a low population density of 513 registered inhabitants, predominantly of the Trio tribe. Indigenous tribes in the interior are ruled by traditional authorities with a socio-cultural and administrative role addressing the day-to-day issues within their territory and coordinating activities with the central Government [13]. Road infrastructure is not available, and boats (dugout canoes) and small airplanes are the main mode of transportation. Housing conditions in this area are traditional, with inhabitants sleeping in hammocks under wooden stilt huts with palm-thatched roofs. There is no access to running water, and electricity is provided solely by diesel generators, and their use depends on the local availability of fuel.

The combination of limited infrastructure and geographical remoteness creates a complex web of logistics challenges to access the population for the delivery of healthcare services.

### Malaria surveillance and response system

The NMP of the Ministry of Health (MoH) maintains the national surveillance database and coordinates malaria activities throughout the country with a focus on mobile migrant populations. Since 2005, the NMP has also spearheaded the implementation of the national malaria elimination strategy with financial support from several international partners, especially the Global Fund to fight AIDS, Tuberculosis and Malaria. The malaria activities of the NMP are complemented by the Medical Mission Primary Health Care Suriname (MM), a non-governmental health organization providing primary health care to remote villages in the interior of the country. All malaria diagnosis, care, treatment and response measures in Suriname are provided free of charge.

The MET is a technical advisory committee to the MoH, composed of malaria experts, with specialized knowledge across various fields. The MET oversees the national malaria elimination effort, guides the national malaria response, and aligns strategies and activities. During the elimination phase, the MET also reviewed each case to assess the best suited response and determined the actual response measures.

Microscopy is the gold standard for malaria diagnosis in Suriname and is a prerequisite for the diagnosis for each case [14]. Since microscopists are not available at all MM clinics in the remote areas for immediate blood smear examination, rapid diagnostic tests (RDTs) are also used in the field and treatment in these areas can be initiated based on a positive RDT result. However, all RDT results are cross-checked with blood smears, as all microscopic slides are sent to the capital (MM Headquarters) either for confirmation after microscopic screening on site or for first reading.

Upon a positive diagnosis or positive RDT, immediate treatment is administered, in accordance with the national malaria treatment guidelines [14]. Since 2009, Dried Blood Spots (DBSs) are collected from all positive cases to enable molecular analysis, when needed, for molecular confirmation of unreliable microscopy results, for differentiation between recrudescence and reinfection, for drug resistance and for outbreak investigations. DBSs are also stored in the National Malaria Gene Bank (NMGB) for future use.

Malaria diagnosis, care and treatment were delivered to the inhabitants of Pelele Tepoe by trained healthcare assistants of MM, with support from the NMP. These healthcare assistants (GZA, *gezondheidszorg assistent*) are often recruited from the local communities and their shared language/dialect, local beliefs and traditions facilitate easy access to care. GZA’s are trained by the MM to follow standardized medical guidelines and protocols for malaria screening with malaria RDTs, preparation and staining of microscopic slides and to provide basic treatment upon phone instructions of the supporting medical doctor. They also gather demographic and epidemiological data, including travel history. GZA’s are supervised during regular clinic visits of the medical doctors assigned to their region and they maintain constant communication with them through telehealth with high frequency short wave radio, smart phones, or satellite phones.

## Results

After a malaria-free period exceeding five years, Pelele Tepoe experienced a *P. vivax* outbreak starting in 2019. The index case was suspected to be a Pelele Tepoe resident who visited Palumeu, a neighboring Amerindian village which had an ongoing *P. vivax* outbreak. The outbreak lasted almost 22 months from Nov 2019 to Aug 2021 and initially 183 infections were registered in 153 patients. Reclassification based on detailed case investigation, travel history and molecular analysis resulted in 179 infections in Pelele Tepoe. Furthermore, among these infections, 176 cases were classified as indigenous outbreak infections, next to 3 imported malaria cases [15].

### Outbreak management approach and measures

Traditional outbreak management strategies and corresponding tools and interventions for 1-3-7 surveillance, community health education and vector control were implemented at the onset of the outbreak. Continuous monitoring of the outbreak trend, examination of each case and thorough evaluation of collected surveillance data guided the selection of additional, less conventional strategies and measures, which were incorporated into the multi-pronged approach. This multi-faceted approach focusing on six key management areas was designed to more effectively contain the outbreak and prevent the reintroduction of malaria in a malaria-eliminating country.

1. “1-3-7” surveillance and response approach

The “1-3-7” surveillance and response strategy for malaria elimination [16] was already adopted by Suriname as surveillance system since 2019.

The numbers 1, 3, and 7 represent the respective deadlines (in days) for each stage of the process. Report a malaria diagnosis within 1 day, complete case investigation within 3 days after reporting, and execute focused investigation and targeted response to prevent further transmission within 7 days.

Although this approach is part of the national malaria prevention and response strategy, the community and local healthcare assistants were not anticipating malaria transmission, due to consecutive years without malaria in Pelele Tepoe. Unfortunately, this misconception resulted in some delays in testing, treatment, and reporting to the NMP at the start of the outbreak. In addition, the screening of migrants and travelers, the timely detection of all malaria cases and implementations of some outbreak measures were negatively impacted by management changes at the MoH and the consequences of the SARS CoV-2 (COVID-19) pandemic.

1. Malaria Health Education and Community Participation

Community engagement had a key role in the implemented awareness program, as increasing malaria knowledge, malaria awareness and community participation are known strategies to keep communities invested and committed to interrupt and prevent malaria transmission. Fostering trust between the MM and the local dignitaries was a vital aspect. In consultation with the community leaders, specifically tailored community awareness sessions, referred to as “Krutu” or traditional community meetings, were organized during the outbreak in November 2019, April, August, October and November 2020, and February, April, August and September 2021. In these meetings, attended by community leaders and villagers, a respectful approach was adopted during the sharing of data, while also encouraging lively interaction with community feedback to evade any unwanted patronizing dynamics. The Information, Education, and Communication (IEC) material regarded contextual malaria information, featuring topics as the causes, transmission, prevention methods, symptoms, testing sites and treatments. The IEC material was reinforced with customized materials such as posters and flyers in the local native language, complemented with vivid illustrations, tailored to the education level of the target population.

The community’s active involvement was evident in the efforts of leaders, who encouraged travelling villagers to test upon returning to the village and advocated for the testing of visiting participants, especially during religious conferences. Additionally, the community was kept regularly updated on the outbreak’s progress through visual displays, including charts showing trends in case numbers. Daily, the total number of positive cases was updated on a writing board at the general meeting site by an appointed community member, after consultation with MM headquarters. Optimized scheduling and execution of visits to the village (only accessible by air) was severely hampered not only by heavy rainfall, which flooded the unpaved grass and sand airstrip throughout the whole rainy season, but also by COVID-19 related lockdowns. Malaria health education and community participation activities were sustained after the outbreak.

1. Vector Control Measures

Another conventional tool during malaria outbreaks are vector control interventions targeting the transmission hotspots to limit the spread of malaria from these high-risk zones to nearby areas. Core vector control interventions such as free of charge provision of long-lasting insecticide-treated nets (LLINs) and mosquito repellant in adult and children’s formulations were deployed throughout the outbreak in collaboration between the MM and the NMP. The local MM health post distributed 840 LLINs, exceeding the village’s population to also allow for old net replacements and provision of LLINs for passing nomad Amerindians.

Furthermore, the community was instructed on the use and washing of LLINs. Also, one round of indoor residual spraying (IRS) with alpha-cypermethrin (Fendona, BASF) was performed on all interior walls and surface areas of each hut, aimed to decrease the density of the adult malaria vectors.

In addition to the aforementioned measures, introduced at the onset of the outbreak, the following prongs were implemented at various stages throughout the outbreak, depending on the evolving needs.

1. Mass Screening and Tailored Treatment

The standard treatment for *vivax* malaria in Suriname consists of Chloroquine 25 mg/kg for 3 days/Primaquine (0.50 mg/kg for 14 days). While Chloroquine (CQ) is effective against the asexual blood stages of *P. vivax*, Primaquine (PQ) tackles the dormant liver-stage parasites (hypnozoites). As the effectiveness of the treatment depends on adherence, all medication was provided as treatment under direct observation therapy (DOT) since the start of the outbreak.

An overview of the different treatment regimens used throughout the outbreak are presented in the Additional file 1: S1 Table.

In line with the country’s policies, ‘accelerator’/reactive strategies were deployed to contain the outbreak.

**Fig 1:**
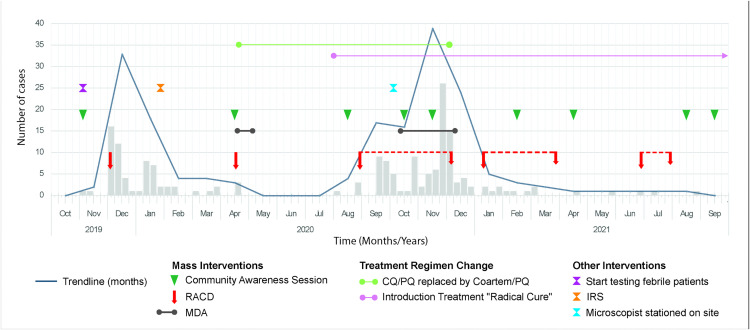
Timeline outbreak cases & interventions.

The timeline in Fig 1 illustrates the outbreak cases as bars for weekly reported cases alongside the implemented interventions.

These measures included intensified malaria screening in response to the local cases, Reactive Case Detections (RACDs) to contain local malaria foci and Mass Drug Administrations (MDAs) to prevent onward malaria transmission, also targeting asymptomatic *P. vivax* carriers.

Starting from November 2019, all febrile patients were tested for malaria by GZA’s from the MM.

The first RACD was implemented four weeks after the first infection, taking into account the parasite incubation period, time lapse for the development of symptoms and logistical challenges at the time, such as transportation and access to remote areas. This RACD reached 448 persons and resulted in 19 additional positive cases.

The second RACD was conducted as the initial outbreak was subsiding, as depicted in Fig 1. The roll-out of an MDA using a Coartem + PQ regimen was also organized, targeting all eligible residents. This MDA reached more than 88.1% of the residing population, with over 95% completing the full course. However, after a 15-weeks hiatus with zero cases, new positive cases were reported, prompting the deployment of a semi-continuous RACD between Aug 29 and December 6, comprising 7 short events, each lasting between one and three days. In addition, a second MDA was implemented, which however had a substantially lower completion rate (68%). In 2021, the persistent but low-level caseload was addressed through semi-continuous RACD efforts as illustrated in Fig 1.

Each MDA reduced the parasite burden in the community as visualized in Fig 1. In addition, a microscopist was stationed at the health post from October 2020 till June 2021 to perform swift microscopic examination of slides, enabling timely response to new cases and allowing the collection and processing of thick smears on site from all participants every two weeks after the second MDA.

Glucose-6-phosphate dehydrogenase (G6PD)-deficiency testing of positive cases was expanded to village wide mapping since April 2020, through on-site G6PD screening with SD BioSensor as G6PD-enzyme activity analyzer. More than 80% of the population could be tested during this endeavor and the overall G6PD deficiency in Pelele Tepoe was estimated at 9.3% (*personal communication dr. S. Vreden*). During the outbreak, just 2 persons were not eligible to receive Primaquine on-site, due to G6PD deficiency.

1. Molecular outbreak reconstruction to guide interventions

In an earlier outbreak in another Amerindian village in Suriname, we demonstrated that molecular genotyping of circulating parasite populations utilizing well characterized polymorphic regions within the gene encoding the merozoite surface protein-3α (Pv*msp-3α*) [17] and merozoite surface protein-1 F2 (*Pvmsp-1 F2*) [18], combined with polymerase chain reaction/restriction fragment length polymorphism (PCR-RFLP) can be a useful tool in regions with low malaria transmission, where the genetic diversity of the parasite population is limited [15].

Therefore, molecular tools for malaria outbreak reconstruction were also utilized in this outbreak to complement the traditional strategies to guide the selection of interventions during this outbreak.

Molecular reconstruction of the outbreak revealed a single clonal lineage, as almost all the tested samples exhibited the same haplotype. The only divergent strains (n = 3) identified during the study [15] were collected from patients with a travel history to Brazil. Their classification as import case was based on the combination of travel history and their genetic profile. The investigated cases with recurring malaria exhibited the same genetic profile. The demonstration of a single clonal lineage during the outbreak enabled the NMP to act accordingly and implement targeted measures such as, allocation of resources to the affected areas, more severe monitoring of travel to and from Pelele Tepoe to other villages in Suriname or neighboring countries and a more stringent screening of travelers. In addition, a more detailed investigation was deployed into the impact of non-Primaquine users on the prolongation of the outbreak, leading to a treatment regimen change aimed at curtailing *P. vivax* relapses.

1. Tackling Malaria Recurrences

The finding of a single clonal lineage throughout the outbreak, accentuated the need to tweak the outbreak control strategies to include interventions, explicitly addressing recurrent infections.

Tackling malaria recurrences can significantly reduce transmission and requires a holistic approach, particularly addressing the challenge posed by *P. vivax*, which can remain dormant in the liver as hypnozoites and then reactivate weeks, months or even years after the initial infection [19].

Adherence to treatment during the outbreak was safeguarded through implementation of direct observation therapy (DOT) for all positive patients during their full course of treatment. The focus in this prong was therefore on the non-use of Primaquine.

According to WHO recommendations [20] and national guidelines, Primaquine was not administered to pregnant or breastfeeding women and infants younger than 6 months of age. PQ was also not provided to G6PD deficient patients, as its potential hemolytic toxicity in these patients, requires close medical supervision during treatment, which is not feasible in the hinterland.

Pregnant or breastfeeding women and G6PD deficient patients with a non-Primaquine-containing antimalarial regimen accounted for 6.5% of the infected villagers. In pregnant or breastfeeding women, even the re-treatment with PQ was delayed in most cases, due to breastfeeding periods or an alternating status between pregnancy and breastfeeding.

During the outbreak, 26 recurrent episodes were recorded in eighteen patients (Table 1). Eleven villagers (n = 11) who had completed a full course of either CQ-PQ or Coartem-PQ treatment under DOT, still experienced recurrent infections, resulting in a recurrence rate of 4.9%, as measured till day 180, which corresponds to a PQ efficacy of 95.1%.

**Table 1:** Primaquine treatment distribution and rate of recurrence.

	Type of Treatment	Total number of patients (n = 153)					Total recurrent episodes during the outbreak	No. Patients with recurrence (day 180)	Rate of recurrence (day 180)
		Patients with Single Infection (n = 135)	Patients with Multiple Infections (n = 18)						
		0 Recurrences (n = 135)	1 Recurrence (n = 13)	2 Recurrences (n = 3)	3 Recurrences (n = 1)	4 Recurrences (n = 1)			
**PQ treated**	No. of patients treated with PQ*(n = 143)	132	10	1	0	0	12	**7**	**4.9%**
**NOT treated with PQ**	**Total No. of patients NOT treated with PQ (n = 10)**	3	3	2	1	1	14	**6**	**60.0%**
	Pregnant/Breastfeeding Patients^#^(n = 8)	*2*	*3*	*2*	*1*	*0*	*10*	***5***	***62.5%***
	G6PD deficient Patients (n = 2)	*1*	*0*	*0*	*0*	*1*	*4*	***1***	***50.0%***

Original infections are not included in the recurrences count.

* Patients received Coartem or Chloroquine co-administered with Primaquine.

# Five Pregnant or breastfeeding patients could still receive PQ within 2 months post infection and are included in the category “treated with PQ”.

G6PD deficiency was identified in two villagers infected with malaria and resulted in their treatment with only CQ (Table 1). One G6PD-deficient patient had a single infection, while the other patient experienced five malaria episodes during the outbreak, with just a nine-month interval between the first and last infection. One month after the fifth episode, this patient was brought to the capital and CQ/PQ treatment was administered under supervision to monitor any potential hemolysis induced by Primaquine.

The recurrence rate by day 180 was significantly higher in the non-PQ category, even reaching 60.0%, compared to just 4.9% in the patients who received PQ (p-value is < 0.001). The difference became even more striking when not just single relapses, but multiple recurrences were taken into account (Table 1). Four individuals in the non-PQ category experienced multiple recurrences and even one person in the PQ category endured two recurrent episodes.

The number of multiple recurrences varied between 2–4 recurrent episodes during the outbreak (Table 1) and the periods between multiple recurrences ranged from 1 to 5 months.

More than half of the recurrent malaria episodes originated from just the few PQ-ineligible individuals, triggering additional interventions and subsequently even adjustment of the treatment regimen. The MET recommended strict follow-up of pregnant or breastfeeding women and G6PD deficient patients, with biweekly microscopic monitoring and prompt full treatment (CQ/PQ) upon concluding of breastfeeding.

As relapses seemed to fuel the outbreak, a new re-treatment protocol was introduced as “Radical Cure” (“cura radical”; CR) for the PQ-treated subpopulation in August 2020, consisting of a full re-treatment with CQ/PQ for all diagnosed Category I cases, six weeks after the end of initial treatment (Additional file 1 S1 Table).

## Discussion

The implementation of a six-pronged strategy to manage the outbreak in Pelele Tepoe proved effective; it successfully contained the outbreak, prevented its spread to nearby villages and curtailed the resurgence of new foci. Additionally, the strains from the three import cases from Brazil remained confined to those patients, highlighting the effectiveness of the monitoring of villagers’ movements.

Some of the implemented interventions deserve particular attention, due to their significance in the six-pronged approach. Raising community awareness through a combination of educational campaigns, targeted promotional materials, and culturally appropriate community involvement, along with real-time updates on the outbreak’s progress, proved to be an effective strategy as was also demonstrated by others [21]. Empowered leaders took the initiative to encourage their villagers to actively participate in proactive measures and insisted on malaria screening for all visitors entering the village, regardless of symptoms. The villagers were highly engaged, as evidenced by their impressive participation in the MDA, with more than 95% completing the full 12 weeks of treatment.

Malaria health education efforts were sustained, and the boosted community awareness remained evident long after the outbreak. In 2023, a group of 14 villagers returned from Brazil and upon entering Suriname, they immediately presented themselves at the first health post for malaria screening, despite their arrival in the evening after an exhausting days-long journey. The screening of the whole group resulted in the prompt diagnosis and treatment of one positive case, preventing onward transmission and averting a potential new outbreak in 2023.

In contrast to some earlier reports contesting the use of RDTs as diagnostic tool [22], the decision to use RDTs was an important factor in achieving timely diagnosis deep in the interior, as it circumvented the unfeasible requirement for microscopy expertise in every small settlement. Treatment could be directly initiated, based on a positive RDT result, underscoring the notion of RDT as game changer in malaria management [23]. It should be noted that all diagnoses were still verified through microscopy, allowing for therapy adjustments if necessary.

Mass screen-and-treat interventions were substantially less effective in reducing parasite prevalence than the MDA interventions, consistent with previous studies [24].

In line with earlier findings regarding the importance of analyses of genetic patterns in *P. vivax* outbreak populations [25, 26], molecular testing during this outbreak revealed the circulation of a single clonal lineage. This key insight allowed the MET/NMP to make informed decisions and implement more precisely tailored intervention strategies such as addressing recurrences, which significantly improved the efficacy of outbreak management.

Suriname also pioneered the mapping of the G6PD status of indigenous communities, prior to the onset of illness. The availability of the G6PD status for at-risk populations offers a clear advantage in controlling future outbreaks, as it ensures timely, widespread, and medically safe administration of Primaquine.

Another innovative asset was the continuous evaluation and need-based adaptation of treatment protocols, resulting in the implementation of an effective re-treatment practice, counteracting malaria relapse cases in non-PQ eligible patients.

However, despite the use of a multi-pronged approach, malaria outbreak control and containment in Pelele Tepoe was challenging as the outbreak occurred against a backdrop of two key contextual events, the COVID-19 pandemic and crucial replacements on senior management level at the Ministry of Health, due to political shifts. The COVID-19 pandemic caused global disruptions in outbreak detection and control activities for non-COVID-19 diseases, as was reported by WHO [27] and others [28]. In Suriname, the outbreak management measures were hampered by the anticipated pandemic consequences as attention and resources were diverted away from malaria to COVID-19 activities. The malaria program and MM experienced a shortage of skilled healthcare personnel, due to their reassignment to COVID-19 care. The psychological distress among health workers and villagers, triggered by fear of the increased likelihood of direct exposure to the SARS CoV-2 virus caused constraints on community awareness activities and interventions such as the stationing of a microscopist, ACD, RACDs and MDAs. The COVID-19-related lockdowns further delayed and restricted some of these activities. A notable consequence in 2020 was the temporary shift in the first-line treatment from CQ/PQ to a combination of artemether-lumefantrine (Coartem) and PQ (Fig 1). Suriname reserved CQ for the treatment of COVID-19 patients in the early months of the pandemic, just like several countries [29], following early *in vitro* studies suggesting its antiviral and anti-inflammatory activity against SARS-CoV-2. This drug switch was not expected to affect the malaria incidence; however, surveillance and outbreak control activities were nonetheless delayed since re-training of GZAs was required. Treatment seeking behavior was also affected, as febrile patients postponed visits to health facilities out of fear for exposure to COVID-19 or unsubstantiated fear of getting vaccinated without their knowledge, which negatively impacted early malaria detection and treatment.

In 2020, changes in leadership and senior management at the Ministry of Health brought new perspectives and shifting health priorities. Their limited understanding of the malaria situation in the country, coupled with the prioritization of different health issues, temporarily affected not only ongoing malaria activities, including border screening of migrants and travelers, but also delayed the response time for some of the scheduled outbreak interventions. Fortunately, the MET as a non-political technical board swiftly engaged newly installed government officials, thus easing the transition, facilitating continuity and minimizing further disruptions.

Collectively, these complex issues negatively impacted the effectiveness and timeliness of “1-3-7” surveillance and response efforts.

Other factors, although anticipated, such as wavering motivation of health workers throughout the lengthy outbreak, the remoteness of the village (hard-to-reach area), logistical challenges, further compounded by bad weather, also influenced the containment of the *P. vivax* outbreak in Pelele Tepoe.

Another key factor was unregistered human movement. Travel without proper screening was presumed to be the cause of the *P. vivax* outbreak in the Amerindian village Palumeu, earlier in 2019, and uncontrolled travel to and from Palumeu appeared to have been the trigger [15] for the Pelele Tepoe outbreak, described here. However, population mobility has played a historic role in the spread of malaria [30] and the mobility from village to village among the Amerindian population in the Guianas, either as cross-border traffic to Brazil, Guyana, and French Guiana or in-country travel between villages, is a fundamental aspect of their nomadic identity [31].

The lessons learned prompted the country to implement a more robust surveillance system to counteract this mobility-induced transmission. The focus was on intense community involvement to endorse traveler screening immediately upon arrival in a village and vigilant GZAs, even in the absence of active cases, through continuous malaria education and microscopy training. The introduction of an enhanced surveillance system resulted in successful early detection and prompt response to malaria cases imported via travelers, as demonstrated by the absence of new outbreaks beyond Pelele Tepoe.

Declining motivation among health workers, particularly during the COVID-19 period, led to a decrease in productivity and less efficient dissemination of information, which was mitigated by the introduction of targeted support programs, digital training platforms, incentives such as food packages, and improved communication strategies. Additionally, regular training sessions implemented after the outbreak ensured that GZAs remained updated with the latest guidelines on screening, testing, reporting, and treating malaria. The NMP/MM persevered in the implementation of health education campaigns and community participation programs, even when severe weather conditions worsened the already challenging access to the remote village, as heavy rains flooded the airstrip disrupting operations and the absence of air transport further complicated the logistics of some pre-scheduled interventions.

Another major challenge in containing the outbreak was the occurrence of *P. vivax* relapses, driven by the reactivation of hypnozoites and primarily attributed to either treatment failure due to antimalarial drug resistance (CQ and/or PQ), or non-compliance with drug regimens, or the non-use of PQ therapy in patients, who were ineligible.

Although resistance of *P. vivax* to CQ has already been documented in some South American countries, including neighboring Guyana [32] and Brazil [33], there have been no clinical indications of Chloroquine-resistant *P. vivax* strains in Suriname. Several studies, especially in Southeast Asia, have documented Primaquine failure despite proper dosing and adherence. The determined PQ efficacy during this outbreak for patients who had completed a full course of CQ-PQ treatment under direct observation therapy was 95.1%, which is in line with the globally reported range of relapses (2.8%–19.0%) in case of correctly administered Primaquine treatment [34, 35]. Concurrent with the expectations, the relapse rate in the non-PQ eligible group was significantly higher than in the PQ treated group and the observed 60% recurrence rate at day 180 was comparable with the recurrence rates in patients treated without Primaquine, as reported in other studies [36].

In the case of multiple recurrent episodes in a patient on PQ therapy, factors other than Chloroquine resistance, low Primaquine doses or a lack of adherence should be considered. The possibility of therapeutic failure, due to impaired metabolism of Primaquine, either by decreased activity of cytochrome P450 2D6 (CYP2D6) [37] or the presence of comorbidities should not be overlooked.

The implementation of the “Radical Cure” for all recurrent patients’ to halt relapses from fueling the outbreak successfully marked the end of the outbreak.

## Conclusion and recommendations

In regions like Suriname, which have the intrinsic potential for malaria transmission due to factors such as a history of high malaria endemicity, favorable climatic conditions for vector breeding, hard-to-reach target populations, porous borders, and ongoing uncontrolled movement of people between neighboring countries with a higher malaria incidence, the resurgence of malaria remains a major concern, as witnessed in various other countries [38].

An integrated, inclusive, and targeted six-pronged approach involving all stakeholders; government health agencies, non-governmental organizations, community leaders, healthcare providers, and international partners, proved essential for effective malaria outbreak management in Pelele Tepoe. We have outlined specific suggestions for regular outbreak approaches and included some non-standard recommendations for regions striving for malaria elimination, with the aim of ensuring sustained progress and preventing setbacks in their elimination efforts.

Intensifying commitment and fostering effective communication between healthcare providers, public health officials, and the community during an outbreak is of paramount importance for facilitating the dissemination of information to the community and for enabling timely adjustments to strategies and interventions. The cornerstone of a successful and sustainable approach to engaging indigenous communities is active consultation on culturally appropriate methods for designing and delivering information and sharing leadership with community leaders, addressing their specific needs and challenges.

In addition, continuous provision of (refresher) training and awareness building for health personnel, partners, and stakeholders, especially through post-outbreak outreach is a prerequisite to maintain vigilance about malaria, preventing misconceptions and avoiding complacency in the execution of program activities. Maintaining periodic community awareness sessions for indigenous and migrant populations, even in post-outbreak situations, will significantly reduce the risk of reintroducing malaria.

Another recommendation is the use of molecular tools in outbreak investigations, as was also advocated in the previous outbreak [26]. The molecular reconstruction of this outbreak implicated relapses as driver of malaria transmission, enabling data-driven decisions, which guided the implementation of a more effective response to contain recurrent malaria. The implementation of effective re-treatment practices, for instance to address malaria relapses are an evident example of the need for continuous evaluation and need-based adaptation of treatment protocols.

The introduction of G6PD pre-screening of at-risk communities is recommended, as it has proven beneficial in administering the complete CQ-PQ treatment in a timely manner, thereby reducing relapses. It has also paved the way for the future optimal use of Tafenoquine (TFQ), as an alternative for PQ in the prevention of *P. vivax* relapses. Single-dose TFQ co-administered with CQ has similar efficacy compared to PQ plus CQ [39], but TFQ has a considerable advantage over the 14-day PQ-regimen in terms of adherence to treatment. Although, adherence to PQ treatment during this outbreak was not an issue, due to DOT, Suriname has already committed to the introduction of TFQ, as DOT may not always be a feasible option, in particular with highly mobile migrants.

Instrumental to the success was the presence of a national Malaria Elimination Task Force, not only to guide the country’s malaria strategy for elimination and prevention of reintroduction, but also to align the response among all actors and provide oversight of the activities of the coordinated outbreak response. While the WHO recommends countries to establish such a national team [40], we specifically advocate for a team composed of multidisciplinary malaria experts, operating independently of government or political pressure. Countries that establish similar national malaria teams will improve the continuity and sustained effectiveness of malaria elimination programs, as the team can provide oversight of malaria elimination efforts, mediate with political leaders and government officials, and actively work to accelerate stakeholder engagement. Furthermore, this team can facilitate smooth transitions in leadership by providing stability and reinforcing political commitment during changes in senior management.

Despite our current malaria elimination achievements, our long-term success hinges on the regional elimination of malaria, as the threat of reintroduction through imported cases remains evident [41], given the significant role of human movement in the spread and reintroduction of malaria, which is further accentuated by the current high endemicity in Guiana Shield countries like Guyana and Venezuela [42]. We strongly advocate for the establishment of regional cooperation programs, fostering partnerships and creating a network to share information and expertise, capable of effectively coordinating collaborative efforts, to implement sustainable malaria control measures across the region.

## Supporting information

S1 Table Treatment regimens. (DOCX)

## Abbreviations

ACD: Active case detection
RACD: Reactive Case Detection
MDA: Mass Drug Administration
CQ: Chloroquine
PQ: Primaquine
TFQ: Tafenoquine
DBS: Dried Blood Spot
DOT: Direct Observation Therapy
G6PD: Glucose-6-Phosphate Dehydrogenase
GZA: Healthcare Assistant
IEC: Information, Education, and Communication
IRS: Indoor Residual Spraying
LLINs: Long-Lasting Insecticide-Treated Nets
MET: Malaria Elimination Taskforce
MM: Medical Mission, Primary Health Care Suriname
MoH: Ministry of Health
NMGB: National Malaria Gene Bank
NMP: National Malaria Program
PCR-RFLP: Polymerase Chain Reaction/Restriction Fragment Length Polymorphism
CYP2D6: Cytochrome P450 2D6
*Pvmsp-1* F2: Merozoite Surface Protein-1 F2 gene of *Plasmodium vivax*
*Pvmsp*-3α: Merozoite Surface Protein-3α gene of *Plasmodium vivax*
RDT: Rapid Diagnostic Test
SDGs: Sustainable Development Goals
WHO: World Health Organization
