# Supplementary material for: A six-pronged approach to manage a Plasmodium vivax outbreak in a low- and middle-income country on the road to malaria elimination
Source: PLoS Negl Trop Dis. 2026 Feb 13;20(2):e0013970. doi: 10.1371/journal.pntd.0013970 (PMC12904374; doi:10.1371/journal.pntd.0013970)
Supplement: S1 Table — (DOCX) [file pntd.0013970.s001.docx]

**S1 Table. Treatment Regimens Pelele Tepoe**

| **#** | **Treatment Type** | **Patient type** | **Period** | **Treatment Regimen**** | **When to start** |
| --- | --- | --- | --- | --- | --- |
| 1 | First line treatment for *P. vivax* **Category I Patients**  (=PQ eligible) | Treatment of choice for uncomplicated *P. vivax* malaria in PQ-eligible patients in Suriname | 4/11/2019 – 16/4/2020 4/12/2020 – Present | CQ + PQ | upon diagnosis |
| 2 | Adapted first line treatment **Category I Patients** | Treatment switch during COVID-19 months (specified period) for uncomplicated *P. vivax* malaria in PQ-eligible patients in Suriname | 17/4/2020 – 3/12/2020 | Coartem® + PQ | upon diagnosis |
| 3 | Pregnant/Breastfeeding/  G6PD* Deficient  **Category II Patients**  (=PQ ineligible) | PQ-ineligible patients | throughout the outbreak | CQ or Coartem® | upon diagnosis |
| 4 | Re-treatment **A** | *P. vivax* negative formerly pregnant or breastfeeding PQ-eligible women with earlier treated infection (Category II patients) | throughout the outbreak | CQ + PQ or  Coartem® + PQ | 6 months after delivery |
| 5 | Re-treatment **CR**  (**"Cura Radical"**) | *P. vivax* negative PQ-eligible patients with earlier treated infection | Aug 2020 – Present | CQ + PQ | six weeks after last day of 1^st^ treatment |
| 6 | Prophylaxis Mass Drug Administration (**MDA**) | All consenting PQ-eligible persons | 15/4/2020 – 5/5/2020 3/10/2020 – 8/12/2020 | Coartem® + PQ | during the MDA |

*Glucose-6-phosphate dehydrogenase

** Chloroquine [25 mg/kg body weight over a 3-day period]

Coartem [Artemether 20 mg/kg body weight and Lumefantrine120 mg/kg body weight, twice daily, for 3 days]

Primaquine [0.50 mg/kg body weight for 14 days]

Pediatric CQ [15 mg/mL]/kg body weight over a 3-day period]

Pediatric PQ [6 mg/5ml]/kg body weight for 14 days]
